# Self-perceived design thinking competence among Taiwanese nursing students: a longitudinal study

**DOI:** 10.1186/s12912-025-04053-1

**Published:** 2025-11-06

**Authors:** Hsing-Yuan Liu

**Affiliations:** 1 Department of Nursing, Chang Gung Institute of Technology, Taoyuan City, Taiwan (R.O.C.); 2 Chang Gung Medical Foundation Linkou, Chang Gung Memorial Hospital, Taoyuan City, Taiwan (R.O.C.)

**Keywords:** Design thinking competence, Nursing education, Longitudinal study, Generalized estimating equations, Self-efficacy theory

## Abstract

**Background:**

Design thinking (DT) is increasingly valued in nursing education for cultivating innovation and problem-solving skills. Although individual differences in DT competence are recognized, the pattern of change in these competencies over time remains unclear.

**Methods:**

Ninety-six nursing students enrolled in a DT training course completed the Creative Synthesis Inventory–Taiwan (CSI–Taiwan) at four time points: before training, immediately after training, and one- and three-months post-training. Students were classified into higher- and lower-scoring groups using K-means clustering. Generalized Estimating Equations (GEE) were used to analyze changes in CSI–Taiwan scores over time.

**Results:**

GEE analysis showed no significant differences in visualization, discovery, prototyping, and evaluation skills immediately after training. Both higher- and lower-scoring students demonstrated significant declines in all DT skills over time. The magnitude of decline was significantly smaller among higher-scoring students, except for the discovery skill between immediately after training and one-month post-training.

**Conclusions:**

These findings align with Bandura’s self-efficacy theory, suggesting that efficacious self-beliefs in DT competence are reflected in differing patterns of change over time. Nursing curricula incorporating longitudinal, spiral models of DT instruction—such as project-based applications in clinical settings—may help consolidate and sustain DT skills, particularly among lower-scoring students.

## Highlights

Less is known whether and how changes would occur in the self-assessment of design thinking (DT) competence across time among nursing students.

DT competence as self-perceived by students was characterized by significant individual differences in the change pattern over a 3-month period after a DT training,

Both higher- and lower-scoring students demonstrated a significant decline over time in DT competence

The magnitude of decline was significantly smaller for the higher-scoring students than the lower-scoring students

It is suggested nursing curricula incorporating longitudinal, spiral models of DT instruction to enhance lower-scoring students’ DT competence.

## Introduction

Design thinking (DT) competence has emerged as a critical capability in nursing education, enabling students to integrate creativity, empathy, and analytical reasoning into patient-centered care. Understanding how students perceive and develop this competence is therefore essential to preparing future nurses for complex practice contexts. As healthcare delivery becomes increasingly complex amid rapid technological and cultural changes, nurses are required to generate innovative solutions to diverse clinical and ethical challenges. Recognizing this need, the American Association of Colleges of Nursing (AACN) identifies DT skills as a core professional competency for preparing future nurses to think critically, collaborate effectively, and adapt to evolving care environments [1]. Since 2008, Tim Brown’s human-centered design concept has revolutionized traditional innovation models, and DT principles have been widely applied to address management and practice problems in healthcare systems [2]. Within higher education, DT has also been adopted as an effective pedagogical framework for fostering creativity and improving learning outcomes, including learning effectiveness [3], motivation [4], innovative thinking [5], and creative self-efficacy [6].

In nursing education, previous studies collectively indicate that incorporating DT into curricula enhances various aspects of student learning, including creative problem-solving, engagement, and clinical reasoning [7, 8]. As a human-centered approach to innovation, DT equips nursing students with essential skills to address complex, real-world challenges in healthcare and patient care [9, 10].

Beyond its general benefits, individual differences in DT competence have also been observed. For instance, Tsai [11] found that Taiwanese female college students scored significantly higher than their male counterparts in overall self-perceived DT competence, while research in Thailand revealed that students with greater prior design experience showed higher DT mindsets following design-based learning [12]. Similarly, Liu [13] identified reliable individual variations among Taiwanese nursing students, noting that those who perceived themselves as less efficacious in creative problem-solving—across visualization, discovery, prototyping, and evaluation—also reported lower confidence and engagement in problem-solving activities. Moreover, both DT competence and self-efficacy are dynamic constructs that evolve with experience, feedback, and instruction [14, 15]. Although self-efficacy development is a central focus in educational research [16–18], little is known about longitudinal changes in DT competence among nursing students. Existing studies have primarily examined changes in self-efficacy within other domains of learning. For example, self-efficacy in mathematics was found to fluctuate across short instructional periods depending on students’ perceived task difficulty and grade level [19], while reading self-efficacy trajectories over 11 months declined among students who experienced fewer social sources of encouragement [20].

In a more recent study, Santos [21] reported that female primary students participating in a DT workshop showed greater gains in self-efficacy than males. To date, only one study has investigated changes in academic self-efficacy among nursing students. Bulfone [22] followed Italian nursing students over three years and found that, although average self-efficacy levels remained stable, significant individual differences emerged by sex, age, and educational background, with females aged 25–50 years and those from scientific fields scoring higher. Collectively, these findings underscore that individual differences and temporal changes in self-efficacy are common across learning contexts, highlighting the need to explore how self-perceived DT competence evolves over time in nursing education.

Despite the absence of a universal definition, DT has been broadly conceptualized as an iterative problem-solving process that involves four key skills—visualization, discovery, prototyping, and evaluation [23]. Visualization refers to the ability to foresee the outcome of an idea or design, while discovery emphasizes understanding users through engagement, observation, feedback, and empathetic listening [24, 25]. Prototyping entails transforming ideas into tangible products or services through iterative testing and refinement, and evaluation involves assessing and improving solutions before implementation [26].

Previous studies with Taiwanese nursing students have verified that the self-assessment of these four DT dimensions is both reliable and valid [13, 27]. From a theoretical standpoint, the concept of competence encompasses not only knowledge and skills but also the confidence to apply them effectively in professional practice. According to Bandura’s self-efficacy theory [14], these self-perceptions of capability influence how individuals approach challenges, persist in difficult tasks, and perform over time. Accordingly, self-perceived DT competence can be viewed as an integration of professional knowledge, creative problem-solving skills, and self-efficacy beliefs that are dynamic and develop through experience and reflection [7, 28, 29]. While previous studies have predominantly employed cross-sectional designs to assess short-term learning outcomes, little is known about how DT competence develops or changes over time. Guided by competence and self-efficacy theories, this study aimed to fill this gap by examining whether and how individual differences in nursing students’ self-perceived DT competence evolve over time.

As reviewed above, students’ self-perception of DT competence can be conceptualized as self-efficacious beliefs in creative problem-solving skills. From this theoretical perspective, nursing students’ DT competence—indexed by the creative problem-solving dimensions of visualization, discovery, prototyping, and evaluation—is expected to be malleable and to show individual differences in patterns of change over time.

Building on this framework, nursing students were selected as the focus of this study because they represent a critical population in which design thinking (DT) competence can be most effectively cultivated during professional training. DT has been increasingly recognized as a core component of nursing curricula that fosters creativity, empathy, and problem-solving skills essential for patient-centered care [1, 6]. Understanding how nursing students perceive and develop DT competence provides valuable insights for designing educational strategies that enhance critical thinking, innovation, and reflective practice. Moreover, examining nursing students’ self-perceived DT competence contributes to evidence-based curriculum development, ensuring that future nurses are well equipped to address complex and evolving healthcare challenges.

### Aims of study

This study aimed to examine longitudinal changes in nursing students’ self-perceived design thinking (DT) competence and to explore individual differences in these changes over time. Specifically, it investigated how students’ creative problem-solving skills—visualization, discovery, prototyping, and evaluation—evolved following DT training and how these trajectories differed between higher- and lower-scoring students.

To strengthen conceptual clarity, this study was guided by three main objectives:

(1) To examine longitudinal changes in nursing students’ self-perceived DT competence;

(2) To compare these changes between higher- and lower-scoring students; and.

(3) To interpret these patterns through the lens of Bandura’s self-efficacy theory.

## Methods

### Study design and participants

This study employed a short-term repeated-measures cohort design, with data collected between September 2021 and June 2022 at a science and technology university in northern Taiwan. Participants were assessed at four time points: before training (Time 0), immediately after training (Time 1), one month after training (Time 2), and three months after training (Time 3). Because the intervention aimed to strengthen design thinking (DT) competence, analytic emphasis was placed on post-training changes (Time 1–Time 3) to evaluate the retention and transfer of learning effects beyond the immediate instructional period. Generalized Estimating Equations (GEE) were used to account for correlations among repeated measurements and to provide population-averaged estimates of longitudinal change.

A total of 96 undergraduate nursing students participated in this study. The majority were female (82.3%) with a mean age of 20.74 years (SD = 1.52). Participants were recruited from a sophomore-level capstone course in the nursing program. Inclusion criteria were: (a) enrollment in the nursing program, (b) willingness to participate in the DT training and complete all surveys, and (c) provision of written informed consent. Exclusion criteria included withdrawal from the course or incomplete questionnaire data. All data were collected anonymously.

### Teaching intervention

The design thinking (DT) training program was implemented as an 18-week, four-credit workshop course specifically designed for sophomore nursing students. The primary goal of the course was to cultivate students’ creative problem-solving abilities and teamwork skills through human-centered design activities. The course was structured around five iterative DT modules—Empathy, Define, Ideate, Prototype, and Test— adapted from the model developed by the Hasso Plattner Institute of Design at Stanford University.

In the Empathy and Define phases, students identified user needs through observation and problem-definition exercises. During the Ideate, Prototype, and Test phases, they generated creative ideas, developed prototypes, and refined solutions based on feedback. The final project required students to present their prototype designs to a multidisciplinary panel of seven experts from fields such as clinical nursing, arts, design thinking, student development, and marketing design. These experts provided formative feedback to help students improve their projects.

Course evaluation was based on continuous participation, teamwork performance, prototype presentation, and peer feedback. The training aimed not only to enhance students’ knowledge and application of design thinking methods but also to strengthen their self-efficacy in addressing nursing-related challenges through innovative, human-centered solutions.

On the first day of training (Time 0), participants completed a questionnaire assessing their DT competence. The same questionnaire was administered three additional times: on the last day of training (Time 1, immediately post-training), one-month post-training (Time 2), and three-months post-training (Time 3).

#### Study sample size calculation

The G*Power program was used to estimate the sample size for an F-test with two between-subjects’ factors, three within-subjects’ factors, and five measurements on the basis of a medium effect size of 0.3, a significant level at α of 0.05, and power (1-β) of 0.8. The estimated minimal sample size was 58. Considering an attrition rate of 20% for repeated-measures studies, the minimum number of students required was 69. The sample size (N) of this study was 96, suggesting a sufficient statistical power.

#### Measurement of the design thinking competence

Students’ design thinking (DT) competence was measured using the Creative Synthesis Inventory – Taiwan version (CSI–Taiwan), which was specifically developed and psychometrically validated for Taiwanese nursing students by Liu [13, 27]. The instrument consists of 20 items rated on a five-point Likert scale ranging from 1 (strongly disagree) to 5 (strongly agree), assessing four dimensions: Visualization (5 items; transforming concrete realities into abstract ideas and integrating previously separate concepts), Discovery (4 items; generating new ideas), Prototyping (4 items; exploring feasibility and developing mock-ups), and Evaluation (6 items; refining solutions and assessing outcomes). The CSI–Taiwan has demonstrated excellent internal consistency (Cronbach‘s α = 0.93) and sound construct validity in prior studies.

In the present study, the previously validated structure was adopted and no additional confirmatory factor analysis (CFA) was conducted, consistent with prior psychometric research on this instrument.

Students completed the CSI–Taiwan at four time points: before training (Time 0), immediately after training (Time 1), one-month post-training (Time 2), and three months post-training (Time 3). Higher scores indicated greater self-perceived DT competence.

Although no additional CFA was performed on the current dataset, the CSI–Taiwan has been rigorously validated in comparable nursing student samples, and this reliance on existing validity evidence is acknowledged as a methodological limitation of the study.

### Statistical analysis

Data analysis was conducted with IBM SPSS Statistics, developed by IBM Inc. in Armonk, NY, USA (version 27.0). Descriptive statistics were employed to describe the demographic characteristics of participants and summarize their DT competence. Pearson’s correlation coefficients were computed to reveal the cross-time correlations within each DT component score and the total score. Additionally, following the study by Liu [27], a K-Means cluster analysis was performed to reveal individual differences in students’ pre-training DT competence. Subsequently, a series of generalized estimating equations (GEE) analyses were performed to explore the change in DT competence among students differing in their pre-training DT competence over time.

To account for the likelihood that the measurements from the same students were correlated, GEE analysis approach is appropriate for the present study [30]. In the GEE analysis, the demographic variables of student age and sex were first included in the models as covariates. Preliminary results showed that neither was significant. As a result, they were removed from all the tested models. The GEE analysis was conducted with the four CSI component scores and their total score as the dependent variable and Time (i.e., Time_1_, Time_2,_ and Time_3_) and Group (i.e., higher- and lower-scoring students revealed by the cluster analysis, with lower-scoring group as the reference) as the independent variables. In addition, the interaction between Time and Group was also included in the models.

### Ethical considerations

The study protocol was approved by the Chang Gung Medical Foundation Institutional Review Board (IRB No. 201800212B0C602). All participants provided written informed consent prior to participation, were assured that their responses would remain anonymous, and were informed of their right to withdraw from the study at any time without penalty.

## Results

### Descriptive statistics and bivariate correlations

The majority of the 96 participants in this study were female (82.3%), and the mean age for the entire sample was 20.74 years (SD = 1.52). Details of the descriptive statistics, including means and standard deviations for the four component and total scores of design thinking (DT) competence as measured by the Taiwanese version of the Creative Synthesis Inventory (CSI), at each of the three post-training time points are shown in Table 1.

**Table 1:** Descriptive statistics for the lower- and higher-scoring students’ design thinking competence as measured by the Taiwanese version of the creative synthesis inventory (CSI-Taiwan) at three time points (*N* = 96)

CSI-Taiwan;	Mean ± SD								
	Time_1_	Time_2_	Time_3_	Time_1_		Time_2_		_Time3_	
	Overall			Lower-scoring students	Higher-scoring students	Lower-scoring students	Higher-scoring students	Lower-scoring students	Higher-scoring students
Visualization	3.92 ± 0.74	3.81 ± 0.69	3.62 ± 0.83	4.01 ± 0.72	3.87 ± 0.75	3.46 ± 0.71	3.86 ± 0.67	3.21 ± 0.70	3.88 ± 0.81
Discovery	4.07 ± 0.72	4.00 ± 0.70	3.65 ± 0.87	4.09 ± 0.67	4.05 ± 0.75	3.61 ± 0.67	3.97 ± 0.78	3.26 ± 0.80	3.89 ± 0.83
Prototyping	3.93 ± 0.72	3.86 ± 0.79	3.65 ± 0.84	3.97 ± 0.73	3.90 ± 0.72	3.46 ± 0.70	3.99 ± 0.71	3.23 ± 0.78	3.91 ± 0.78
Evaluation	3.99 ± 0.74	3.89 ± 0.80	3.65 ± 0.88	4.02 ± 0.65	3.97 ± 0.80	3.60 ± 0.65	3.98 ± 0.80	3.25 ± 0.81	3.90 ± 0.84
Total	4.05 ± 0.63	3.99 ± 0.59	3.70 ± 0.74	4.05 ± 0.60	4.05 ± 0.65	3.56 ± 0.50	4.15 ± 0.57	3.28 ± 0.67	3.96 ± 0.66

The sample size for the lower-scoring students was 37 and higher-scoring students was 59, Time_1_ immediately post-training; Time_2_ 1-month post-training, Time_3_ 3-month post-training

The sample consisted of 37 lower-scoring and 59 higher-scoring students. DT competence was measured immediately post-training (Time1), one-month post-training (Time2), and three months post-training (Time3). Pearson’s correlation analyses were conducted to examine cross-time correlations for the component and total scores of students’ DT competence. The significant cross-time correlations for the four components are summarized as follows:

- Visualization: correlations between Time1–Time2 (*r* = 0.422; *p* < 0.001), Time1–Time3 (*r* = 0.342; *p* < 0.001), and Time2–Time3 (*r* = 0.380; *p* < 0.001) were all positive.
- Discovery: correlations between Time1–Time2 (*r* = 0.339; *p* < 0.001), Time1–Time3 (*r* = 0.460; *p* < 0.001), and Time2–Time3 (*r* = 0.293; *p* < 0.005) were all positive.
- Prototyping: correlations between Time1–Time2 (*r* = 0.324; *p* < 0.001), Time1–Time3 (*r* = 0.845; *p* < 0.001), and Time2–Time3 (*r* = 0.315; *p* < 0.005) were all positive.
- Evaluation: correlations between Time1–Time2 (*r* = 0.269; *p* < 0.001), Time1–Time3 (*r* = 0.403; *p* < 0.001), and Time2–Time3 (*r* = 0.332; *p* < 0.001) were all positive.

For total scores, positive correlations were also observed between Time1–Time3 (*r* = 0.540; *p* < 0.001) and Time2–Time3 (*r* = 0.419; *p* < 0.001).

Overall, these results indicate that DT competence scores were not independent across time points and that correlations were stronger between adjacent assessments.

Therefore, a first-order autoregressive correlation structure was adopted for subsequent GEE analyses.

### Individual differences in pre-training competence (Cluster analysis)

Following Liu [27], K-Means cluster analysis was performed to identify individual differences in students by classifying them into distinct groups based on their total DT competence scores at pre-training. The analysis yielded two clusters: lower-scoring students (*n* = 37) and higher-scoring students (*n* = 59). There were no significant differences in age or gender distribution between the two groups.

### Longitudinal changes in DT competence (GEE analysis)

To examine individual differences in changes in students’ self-perceived DT competence over time, five sets of generalized estimating equations (GEE) analyses were performed. These included the four DT components—visualization, discovery, prototyping, and evaluation—as well as total DT competence. In the GEE models, Group (higher- vs. lower-scoring students), Time (Time1, Time2, Time3), and their interaction (Group × Time) served as predictors. The Group × Time interaction indicated whether the change in scores between two time points differed between the groups.

### Visualization

**Fig. 1:**
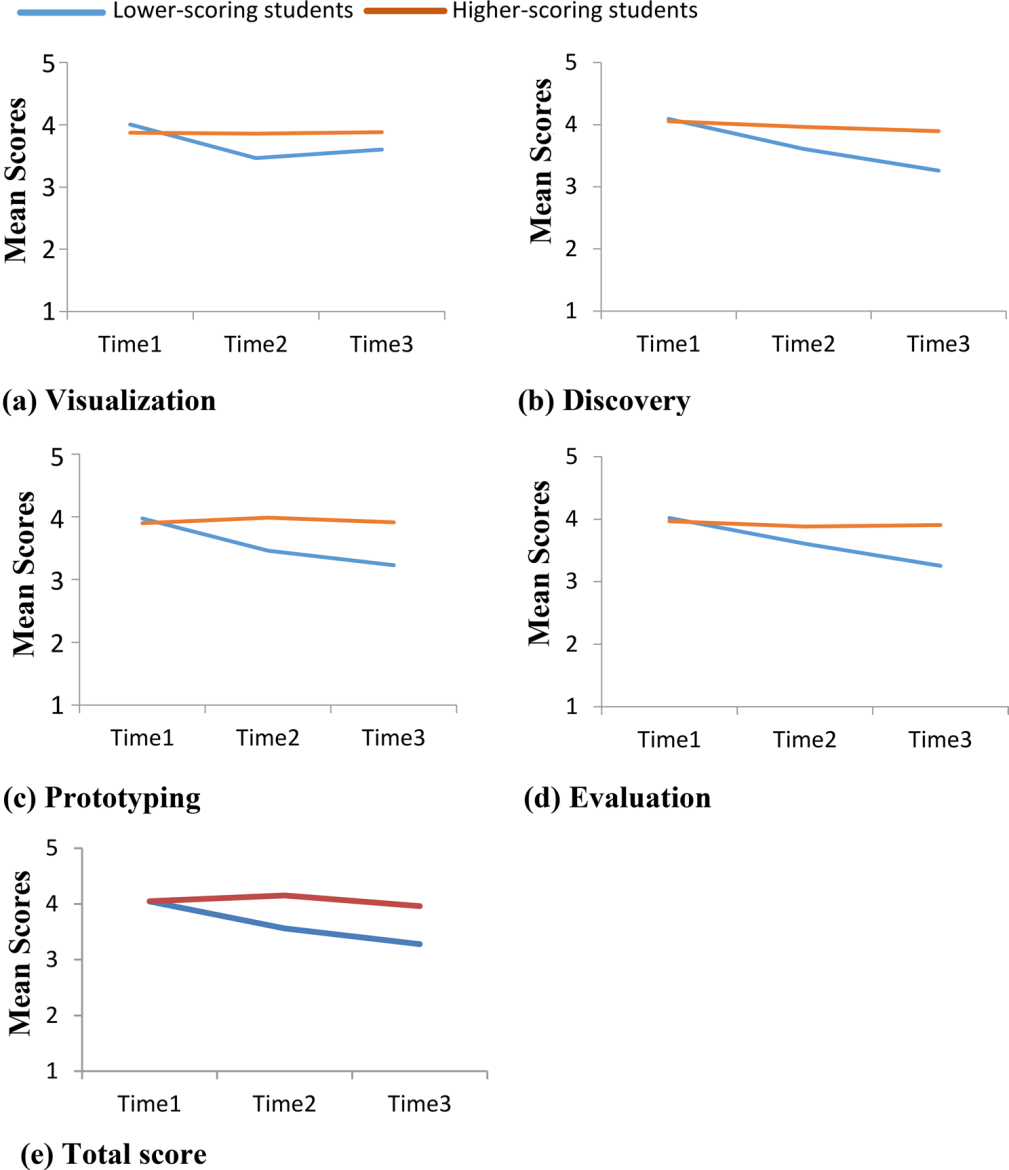
The mean scores of the higher-scoring and lower-scoring students’ design thinking competence as measured by the Taiwanese version of Creative Synthesis Inventory at the three time points of post-training for the component scores of (**a**) visualization, (**b**) discovery, (**c**) prototyping, (**d**) evaluation, and (**e**) the total score, Time1, Time2, Time3: Time_1_, Time_2_, Time_3_; Time_1_: immediately post-training; Time_2_: 1-month post-training; Time_3_: 3-month post-training

No significant group difference was found at Time1. Higher-scoring students exhibited a significantly smaller decrease from Time1 to Time2 (0.541 points) and from Time1 to Time3 (0.795 points) compared with lower-scoring students. Significant Group × Time interactions indicated that higher-scoring students maintained higher scores from Time1 to Time2 (0.527 points) and from Time1 to Time3 (0.805 points). See Table 2 for parameter estimates and Fig. 1a for mean scores.

**Table 2:** Results of generalized estimating equations analysis: the four components of students’ design thinking competence as measured by the Taiwanese version of the creative synthesis inventory (CSI-Taiwan; *N* = 96)

Predictors	CSI-Taiwan											
	Visualization			Discovery			Prototyping			Evaluation		
	β	95% CI	*p*	β	95% CI	*p*	β	95% CI	*p*	β	95% CI	*p*
Group^a^	-0.134	[-0.433, 0.165]	0.379	-0.038	[-0.323, 0.248]	0.796	-0.075	[-0.369, 0.220]	0.619	-0.052	[-0.342, 0.238]	0.725
Time^b^												
Time_2_	-0.541	[-0.862, -0.219]	< 0.001	-0.481	[-0.782, -0.180]	0.002	-0.514	[-0.834, -0.193]	0.002	-0.414	[-0.708, -0.121]	0.006
Time_3_	-0.795	[-1.115, -0.474]	< 0.001	-0.832	[-1.164, -0.501]	< 0.001	-0.746	[-1.085, -0.407]	< 0.001	-0.766	[-1.095, -0.436]	< 0.001
Group x Time												
Higher-scoring students x Time_2_	0.527	[0.117, 0.937]	0.012	0.393	[-0.014, 0.800]	0.058	0.602	[0.192, 1.012]	0.004	0.426	[0.016, 0.835]	0.041
Higher-scoring students x Time_3_	0.805	[0.376, 1.230]	< 0.001	0.673	[0.237, 1.109]	0.002	0.760	[0.327, -1.192]	< 0.001	0.704	[0.263, 1.145]	0.002

*β* parameter estimate, CI confidence interval, ^a^ Reference lower-scoring students, ^b^ Reference Time_1,_ Time_1_ immediately post-training, Time_2_ 1-month post-training, Time_3_ 3-month post-training

### Discovery

Similar to visualization, there was no significant group difference at Time1. Higher-scoring students demonstrated a significantly smaller decline from Time1 to Time2 (0.481 points) and from Time1 to Time3 (0.832 points). A significant Group × Time interaction was observed between Time1 and Time3 (0.673 points), indicating that higher-scoring students maintained higher discovery scores over time. See Table 2; Fig. 1b. Figure 1b.

### Prototyping

No significant difference was found between groups at Time1. Higher-scoring students showed a smaller decrease from Time1 to Time2 (0.514 points) and from Time1 to Time3 (0.746 points) than lower-scoring students. Significant interactions revealed that higher-scoring students achieved higher scores from Time1 to Time2 (0.602 points) and from Time1 to Time3 (0.760 points). See Table 2; Fig. 1c.

### Evaluation

For evaluation, no significant group difference was found at Time1. Higher-scoring students showed a smaller decline from Time1 to Time3 (0.766 points), consistent with the pattern observed in other DT components. These findings indicate that although both groups experienced a post-training decrease in evaluation skills, higher-scoring students maintained relatively higher competence levels over time. See Table 2; Fig. 1d.

### Total scores

Consistent with the findings for the four DT components, no significant difference was observed between groups at Time1. Higher-scoring students exhibited a smaller decline from Time1 to Time2 (0.486 points) and from Time1 to Time3 (0.766 points) compared to lower-scoring students. Significant interactions indicated that higher-scoring students maintained higher total DT scores from Time1 to Time2 (0.588 points) and from Time1 to Time3 (0.675 points). See Table 3; Fig. 1e.

**Table 3:** Results of generalized estimating equations analysis: the total score of students’ design thinking competence as measured by the Taiwanese version of the creative synthesis inventory (*N* = 96)

Predictor	β	95% CI	*p*
Group^a^	0.006	[-0.247, 0.259]	0.964
Time^b^			
Time_2_	-0.486	[-0.736, -0.237]	< 0.001
Time_3_	-0.766	[-1.051, -0.480]	< 0.001
Group x Time			
Higher-scoring students x Time_2_	0.588	[0.255, 0.961]	< 0.001
Higher-scoring students x Time_3_	0.675	[0.305, 1.045]	< 0.001

Abbreviations: *β* = parameter estimate; CI = confidence interval

^a^ Reference = lower-scoring students

^b^ Reference = Time_1_

Note. Time_1_ = immediately post-training; Time_2_ = 1-month post-training; Time_3_ = 3-month post-training

### Summary of findings

Overall, the GEE analyses revealed three main findings:

1. Both higher- and lower-scoring students experienced declines in self-perceived DT competence during the post-training period;
2. The magnitude of decline was consistently smaller among higher-scoring students across all DT components; and
3. The only exception was the discovery component, where the difference between groups was not significant from Time1 to Time2.

These results suggest that students with initially higher DT competence were more likely to sustain their self-perceived creative problem-solving abilities over time.

## Discussion

### Longitudinal changes in self-perceived DT competence

The present study was the first to employ a longitudinal design to investigate nursing students’ self-perceived design thinking (DT) competence over time. The findings revealed that both higher- and lower-scoring students experienced a gradual decline in DT competence following DT training, across all four dimensions—visualization, discovery, prototyping, and evaluation—as well as in the total score. This pattern suggests that while DT-based learning can initially enhance students’ creative problem-solving abilities, its effects may gradually diminish without continued reinforcement. Similar post-training declines have been observed in studies of academic self-efficacy [22, 31], indicating that such competencies are dynamic, context-dependent, and sensitive to motivational factors.

Building on Bandura’s framework, these four sources provide meaningful explanations for the observed post-training decline in DT competence. Specifically, the temporary decrease in students’ self-perceived competence may reflect reduced mastery experiences once the structured training context ended, fewer opportunities for vicarious learning from peers, and diminished verbal reinforcement from instructors. Moreover, increased emotional and physiological stress during clinical courses may have contributed to a lower sense of creative efficacy. Taken together and interpreted through Bandura’s lens, these findings suggest that changes in perceived competence are not merely cognitive fluctuations but are shaped by contextual and affective factors over time.

The decline in Taiwanese nursing students’ self-assessed DT efficacy after training may therefore be explained by the lack of positive reinforcement in one or more of these influential sources. However, beyond Bandura’s framework, several contextual and situational factors may also account for the observed post-training decline in self-perceived DT competence. First, the fading effect of short-term interventions is well documented [31, 32], suggesting that without continuous reinforcement, learners’ confidence in applying newly acquired skills tends to diminish over time. Second, increased academic or clinical workload may have reduced students’ perceived creative efficacy [33, 34]. Third, repeated administration of the same questionnaire may have led to assessment fatigue, reducing engagement and attentiveness during self-assessment [35, 36]. Collectively, these factors provide complementary explanations that extend beyond self-evaluation bias and highlight the importance of sustained, practice-integrated approaches for maintaining design-thinking competence.

### Individual differences in DT competence trajectories

The second key finding demonstrated consistent individual differences in the rate of change between higher- and lower-scoring students. Although both groups exhibited post-training declines, higher-scoring students showed a smaller magnitude of decline across most DT components, with the exception of the discovery skill between immediate and one-month post-training. This pattern aligns with competence and self-efficacy theory, which suggest that learners with stronger initial beliefs in their abilities are more resilient and maintain higher motivation under challenging conditions.

Differences in self-assessment bias may further explain these findings. Higher-scoring students may be more prone to a self-serving bias—the tendency to overestimate their competence—thus maintaining elevated self-perceptions even as actual ability stabilizes [37]. Conversely, lower-scoring students may display a modesty bias—the inclination to underestimate their capabilities and avoid high self-ratings [20, 29, 37, 38]—which may contribute to a steeper decline in perceived competence.

Interestingly, the absence of a significant between-group difference for the discovery dimension suggests that both groups possessed relatively stable self-efficacy in this domain. Prior research found that Taiwanese nursing students tend to rate themselves higher in discovery than in other DT skills [27], possibly due to greater confidence in their ability to observe, empathize, and identify user needs—core strengths of the nursing profession. Although the present study examined individual differences through two baseline groups, this approach provides an initial yet limited view of within-person variability. Future research employing more advanced longitudinal or growth modeling methods could yield deeper insights into the dynamic trajectories of DT competence development.

### Educational and theoretical implications

These findings underscore the importance of maintaining DT competence through continuous reinforcement within nursing curricula. The short-term gains observed immediately after training highlight the need for longitudinal curricular integration—embedding DT practice across multiple courses, simulation exercises, and clinical contexts.

Repeated opportunities to ideate, prototype, and evaluate solutions can sustain learning motivation and confidence. Educators should also consider differentiated instruction and mentorship to support students with lower self-efficacy. Early identification of students with modest DT confidence could inform targeted interventions that build creative self-beliefs through feedback, reflection, and peer collaboration.

Theoretically, this study supports the conceptualization of DT competence as a multidimensional construct encompassing cognitive, affective, and metacognitive elements that evolve over time. The interaction between self-efficacy and creative confidence observed here reflects the intertwined nature of knowledge, skills, and belief systems in professional competence development.

Future research may explore how feedback mechanisms and learning climate shape the sustainability of DT competence in diverse nursing education settings.

## Conclusions

The present study contributes longitudinal evidence that design thinking (DT) competence, operationalized as efficacy beliefs in creative problem-solving, is malleable and sensitive to initial competence levels. Understanding how students develop competencies over time is essential for evaluating the long-term effectiveness of educational interventions. The findings highlighted important practical implications for nursing education. As theorized by self-efficacy theory [14], self-perceived DT competence may evolve over time with experience, feedback, and instruction [15]. Students with lower initial self-efficacy beliefs may benefit from extended scaffolding, guided reflection, or more frequent opportunities to practice DT skills beyond the initial training period. Nursing educators should consider curricula that revisit and reinforce DT principles. Specifically, incorporating longitudinal, spiral models of DT instruction—such as project-based applications in clinical settings or integration with capstone experiences—may help consolidate and sustain DT skills.

Finally, while this study provides new insights into the temporal dynamics of DT competence among Taiwanese nursing students, several limitations warrant consideration. First, the sample was drawn from a single institution in northern Taiwan, which may limit the generalizability of the findings. Future studies should aim to replicate this research in other geographic regions, institutional types, and cultural contexts.Second, this study solely relied on a self-report instrument on DT competence. Self-assessments may not always align with actual performance and under- or over-estimate biases in self-assessment frequently occur [39]. Therefore, future studies should include actual performance measurement such as students’ grades as the indices for DT competence. Moreover, nursing researchers need to be aware that the potential cognitive biases in self-assessment data, which may result in biased estimates and incorrect conclusions.

## Data Availability

The datasets generated and analyzed during the current study are not publicly available to ensure data confidentiality.
